# Supplementary material for: The use of wireless sensors in the neonatal intensive care unit: a study protocol
Source: PeerJ. 2023 Jun 27;11:e15578. doi: 10.7717/peerj.15578 (PMC10312156; doi:10.7717/peerj.15578)
Supplement: Supplemental Information 3 [file peerj-11-15578-s003.docx]

**DATA COLLECTION FORM (PHASE 1)**

**BASIC STUDY INFORMATION**

Patient ID: _____________

Date and time of study initiation: _____________ (YYYY-MM-DD) ____________ (HH:MM)

Date and time of study completion: _____________ (YYYY-MM-DD) ____________ (HH:MM)

**DEMOGRAPHICS**

| Year of birth: _______ | Sex: ☐ Male ☐ Female | Hours of life (at enrollment): ________ |
| --- | --- | --- |
| Gestational age (at birth): ______ weeks ______ days | | Birth weight: _________ grams |
| Diagnosis(es) at time of enrollment: | | |
| ☐ Perinatal asphyxia ☐ Anemia ☐ Apneas and bradycardias ☐ Bronchopulmonary dysplasia | | |
| ☐ Hydrocephalus ☐ Intraventricular hemorrhage ☐ Jaundice ☐ Necrotizing enterocolitis | | |
| ☐ Other, specify: ______________________________ | | |
| Treatments at enrollment: | | |
| ☐ Therapeutic hypothermia | | ☐ Continuous positive airway pressure (CPAP) |
| ☐ Conventional mechanical ventilation (CMV) | | ☐ High frequency ventilation (HFV) |
| ☐ Nasal intermittent positive pressure ventilation (NIPPV) | | ☐ Not applicable/healthy infants |
| Patient group: ☐ A ☐ B ☐ C ☐ D ☐ E ☐ F ☐ G ☐ H | | |

| A = healthy term infants in room air at enrollment  B = term infants with perinatal asphyxia undergoing therapeutic hypothermia at enrollment  C = healthy preterm infants in room air at enrollment  D = preterm infants on continuous positive airway pressure at enrollment  E = preterm infants on conventional mechanical ventilation at enrollment  F = preterm infants on high frequency ventilation at enrollment  G = preterm infants on nasal intermittent positive end expiratory pressure at enrollment  H = preterm infants on continuous positive airway pressure at enrollment |
| --- |

**HOURLY LOGS**

Day 1:

| Date: Start Time: End Time: | | | | | | | | | |
| --- | --- | --- | --- | --- | --- | --- | --- | --- | --- |
|  | baseline | 1h | 2h | 3h | 4h | 5h | 6h | 7h | 8h |
| Location of chest unit  (Center, R or L side) |  |  |  |  |  |  |  |  |  |
| Location of limb unit (R-right, L-left, H-hand/wrist, F-foot) |  |  |  |  |  |  |  |  |  |
| Number of conventional wires |  |  |  |  |  |  |  |  |  |
| Axillary temperature (°C) |  |  |  |  |  |  |  |  |  |
| Room temperature (°C) |  |  |  |  |  |  |  |  |  |
| Air temperature (°C) & humidity (%) - 15cm from neonate |  |  |  |  |  |  |  |  |  |
| Kangaroo care  (Y/N) |  |  |  |  |  |  |  |  |  |
| Nursing care  (Y/N) |  |  |  |  |  |  |  |  |  |
| Medications  (Write down any medication in use) |  |  |  |  |  |  |  |  |  |
| Phototherapy  (Y/N) |  |  |  |  |  |  |  |  |  |
| Type of ventilatory support (CPAP, CMV, HFV, NIPPV) |  |  |  |  |  |  |  |  |  |
| Neonate location  (incubator/crib) |  |  |  |  |  |  |  |  |  |
| Neonate position  (s-supine, p-prone, side) |  |  |  |  |  |  |  |  |  |
| Sensor disconnection (N)/reasons* |  |  |  |  |  |  |  |  |  |

*A = x-ray, B = bath, C = cleaning, D = tests, E = transport, F = other

Photographs of the skin at sensor placement sites (place “✓” if done)

| Chest | ☐ Baseline (0h) | Limb | ☐ Baseline (0h) |
| --- | --- | --- | --- |
|  | ☐ 8h |  | ☐ 8h |

Neonatal Infant Pain Scale – Day 1

| **Pain Assessment** | | Score | |
| --- | --- | --- | --- |
| **Facial Expression** | |  | |
| 0 – Relaxed muscles | Restful face, neutral expression | | |
| 1 – Grimace | Tight facial muscles, furrowed brow, chin, jaw, (negative facial expression – nose, mouth, brow) | | |
| **Cry** | |  | |
| 0 – No cry | Quiet, not crying | | |
| 1 - Whimper | Mild moaning, intermittent | | |
| 2 – Vigorous Cry | Loud scream; rising, shrill, continuous (Note: silent cry may be scored if baby is intubated as evidenced by obvious mouth and facial movement.) | | |
| **Breathing Patterns** | |  | |
| 0 – Relaxed | Usual pattern for this infant | | |
| 1 – Change in Breathing | Indrawing, irregular, faster than usual, gagging, breath holding | | |
| **Arms** | |  | |
| 0 – Relaxed/Restrained | No muscular rigidity, occasional random movements of arms | | |
| 1 – Flexed/Extended | Tense, straight legs; rigid and/or rapid extension/flexion | | |
| **Legs** | |  | |
| 0 – Relaxed/Restrained | No muscular rigidity, occasional random movements of legs | | |
| 1 – Flexed/Extended | Tense, straight legs; rigid and/or rapid extension/flexion | | |
| **State of Arousal** | |  | |
| 0 – Sleeping/Awake | Quiet, peaceful, sleeping, or alert random leg movement | | |
| 1 – Fussy | Alert, restless, thrashing | | |
| **TOTAL** | | |  |

Rater: ____________

Day 2:

| Date: Start Time: End Time: | | | | | | | | | |
| --- | --- | --- | --- | --- | --- | --- | --- | --- | --- |
|  | baseline | 1h | 2h | 3h | 4h | 5h | 6h | 7h | 8h |
| Location of chest unit  (Center, R or L side) |  |  |  |  |  |  |  |  |  |
| Location of limb unit (R-right, L-left, H-hand/wrist, F-foot) |  |  |  |  |  |  |  |  |  |
| Number of conventional wires |  |  |  |  |  |  |  |  |  |
| Axillary temperature (°C) |  |  |  |  |  |  |  |  |  |
| Room temperature (°C) |  |  |  |  |  |  |  |  |  |
| Air temperature (°C) & humidity (%) - 15cm from neonate |  |  |  |  |  |  |  |  |  |
| Kangaroo care  (Y/N) |  |  |  |  |  |  |  |  |  |
| Nursing care  (Y/N) |  |  |  |  |  |  |  |  |  |
| Medications  (Write down any medication in use) |  |  |  |  |  |  |  |  |  |
| Phototherapy  (Y/N) |  |  |  |  |  |  |  |  |  |
| Type of ventilatory support (CPAP, CMV, HFV, NIPPV) |  |  |  |  |  |  |  |  |  |
| Neonate location  (incubator/crib) |  |  |  |  |  |  |  |  |  |
| Neonate position  (s-supine, p-prone, side) |  |  |  |  |  |  |  |  |  |
| Sensor disconnection (N)/reasons* |  |  |  |  |  |  |  |  |  |

*A = x-ray, B = bath, C = cleaning, D = tests, E = transport, F = other

Photographs of the skin at sensor placement sites (place “✓” if done)

| Chest | ☐ Baseline (0h) | Limb | ☐ Baseline (0h) |
| --- | --- | --- | --- |
|  | ☐ 8h |  | ☐ 8h |

Neonatal Infant Pain Scale – Day 2

| **Pain Assessment** | | Score | |
| --- | --- | --- | --- |
| **Facial Expression** | |  | |
| 0 – Relaxed muscles | Restful face, neutral expression | | |
| 1 – Grimace | Tight facial muscles, furrowed brow, chin, jaw, (negative facial expression – nose, mouth, brow) | | |
| **Cry** | |  | |
| 0 – No cry | Quiet, not crying | | |
| 1 - Whimper | Mild moaning, intermittent | | |
| 2 – Vigorous Cry | Loud scream; rising, shrill, continuous (Note: silent cry may be scored if baby is intubated as evidenced by obvious mouth and facial movement.) | | |
| **Breathing Patterns** | |  | |
| 0 – Relaxed | Usual pattern for this infant | | |
| 1 – Change in Breathing | Indrawing, irregular, faster than usual, gagging, breath holding | | |
| **Arms** | |  | |
| 0 – Relaxed/Restrained | No muscular rigidity, occasional random movements of arms | | |
| 1 – Flexed/Extended | Tense, straight legs; rigid and/or rapid extension/flexion | | |
| **Legs** | |  | |
| 0 – Relaxed/Restrained | No muscular rigidity, occasional random movements of legs | | |
| 1 – Flexed/Extended | Tense, straight legs; rigid and/or rapid extension/flexion | | |
| **State of Arousal** | |  | |
| 0 – Sleeping/Awake | Quiet, peaceful, sleeping, or alert random leg movement | | |
| 1 – Fussy | Alert, restless, thrashing | | |
| **TOTAL** | | |  |

Rater: ____________

Day 3:

| Date: Start Time: End Time: | | | | | | | | | |
| --- | --- | --- | --- | --- | --- | --- | --- | --- | --- |
|  | baseline | 1h | 2h | 3h | 4h | 5h | 6h | 7h | 8h |
| Location of chest unit  (Center, R or L side) |  |  |  |  |  |  |  |  |  |
| Location of limb unit (R-right, L-left, H-hand/wrist, F-foot) |  |  |  |  |  |  |  |  |  |
| Number of conventional wires |  |  |  |  |  |  |  |  |  |
| Axillary temperature (°C) |  |  |  |  |  |  |  |  |  |
| Room temperature (°C) |  |  |  |  |  |  |  |  |  |
| Air temperature (°C) & humidity (%) - 15cm from neonate |  |  |  |  |  |  |  |  |  |
| Kangaroo care  (Y/N) |  |  |  |  |  |  |  |  |  |
| Nursing care  (Y/N) |  |  |  |  |  |  |  |  |  |
| Medications  (Write down any medication in use) |  |  |  |  |  |  |  |  |  |
| Phototherapy  (Y/N) |  |  |  |  |  |  |  |  |  |
| Type of ventilatory support (CPAP, CMV, HFV, NIPPV) |  |  |  |  |  |  |  |  |  |
| Neonate location  (incubator/crib) |  |  |  |  |  |  |  |  |  |
| Neonate position  (s-supine, p-prone, side) |  |  |  |  |  |  |  |  |  |
| Sensor disconnection (N)/reasons* |  |  |  |  |  |  |  |  |  |

*A = x-ray, B = bath, C = cleaning, D = tests, E = transport, F = other

Photographs of the skin at sensor placement sites (place “✓” if done)

| Chest | ☐ Baseline (0h) | Limb | ☐ Baseline (0h) |
| --- | --- | --- | --- |
|  | ☐ 8h |  | ☐ 8h |

Neonatal Infant Pain Scale – Day 3

| **Pain Assessment** | | Score | |
| --- | --- | --- | --- |
| **Facial Expression** | |  | |
| 0 – Relaxed muscles | Restful face, neutral expression | | |
| 1 – Grimace | Tight facial muscles, furrowed brow, chin, jaw, (negative facial expression – nose, mouth, brow) | | |
| **Cry** | |  | |
| 0 – No cry | Quiet, not crying | | |
| 1 - Whimper | Mild moaning, intermittent | | |
| 2 – Vigorous Cry | Loud scream; rising, shrill, continuous (Note: silent cry may be scored if baby is intubated as evidenced by obvious mouth and facial movement.) | | |
| **Breathing Patterns** | |  | |
| 0 – Relaxed | Usual pattern for this infant | | |
| 1 – Change in Breathing | Indrawing, irregular, faster than usual, gagging, breath holding | | |
| **Arms** | |  | |
| 0 – Relaxed/Restrained | No muscular rigidity, occasional random movements of arms | | |
| 1 – Flexed/Extended | Tense, straight legs; rigid and/or rapid extension/flexion | | |
| **Legs** | |  | |
| 0 – Relaxed/Restrained | No muscular rigidity, occasional random movements of legs | | |
| 1 – Flexed/Extended | Tense, straight legs; rigid and/or rapid extension/flexion | | |
| **State of Arousal** | |  | |
| 0 – Sleeping/Awake | Quiet, peaceful, sleeping, or alert random leg movement | | |
| 1 – Fussy | Alert, restless, thrashing | | |
| **TOTAL** | | |  |

Rater: ____________

Day 4:

| Date: Start Time: End Time: | | | | | | | | | |
| --- | --- | --- | --- | --- | --- | --- | --- | --- | --- |
|  | baseline | 1h | 2h | 3h | 4h | 5h | 6h | 7h | 8h |
| Location of chest unit  (Center, R or L side) |  |  |  |  |  |  |  |  |  |
| Location of limb unit (R-right, L-left, H-hand/wrist, F-foot) |  |  |  |  |  |  |  |  |  |
| Number of conventional wires |  |  |  |  |  |  |  |  |  |
| Axillary temperature (°C) |  |  |  |  |  |  |  |  |  |
| Room temperature (°C) |  |  |  |  |  |  |  |  |  |
| Air temperature (°C) & humidity (%) - 15cm from neonate |  |  |  |  |  |  |  |  |  |
| Kangaroo care  (Y/N) |  |  |  |  |  |  |  |  |  |
| Nursing care  (Y/N) |  |  |  |  |  |  |  |  |  |
| Medications  (Write down any medication in use) |  |  |  |  |  |  |  |  |  |
| Phototherapy  (Y/N) |  |  |  |  |  |  |  |  |  |
| Type of ventilatory support (CPAP, CMV, HFV, NIPPV) |  |  |  |  |  |  |  |  |  |
| Neonate location  (incubator/crib) |  |  |  |  |  |  |  |  |  |
| Neonate position  (s-supine, p-prone, side) |  |  |  |  |  |  |  |  |  |
| Sensor disconnection (N)/reasons* |  |  |  |  |  |  |  |  |  |

*A = x-ray, B = bath, C = cleaning, D = tests, E = transport, F = other

Photographs of the skin at sensor placement sites (place “✓” if done)

| Chest | ☐ Baseline (0h) | Limb | ☐ Baseline (0h) |
| --- | --- | --- | --- |
|  | ☐ 8h |  | ☐ 8h |

Neonatal Infant Pain Scale – Day 4

| **Pain Assessment** | | Score | |
| --- | --- | --- | --- |
| **Facial Expression** | |  | |
| 0 – Relaxed muscles | Restful face, neutral expression | | |
| 1 – Grimace | Tight facial muscles, furrowed brow, chin, jaw, (negative facial expression – nose, mouth, brow) | | |
| **Cry** | |  | |
| 0 – No cry | Quiet, not crying | | |
| 1 - Whimper | Mild moaning, intermittent | | |
| 2 – Vigorous Cry | Loud scream; rising, shrill, continuous (Note: silent cry may be scored if baby is intubated as evidenced by obvious mouth and facial movement.) | | |
| **Breathing Patterns** | |  | |
| 0 – Relaxed | Usual pattern for this infant | | |
| 1 – Change in Breathing | Indrawing, irregular, faster than usual, gagging, breath holding | | |
| **Arms** | |  | |
| 0 – Relaxed/Restrained | No muscular rigidity, occasional random movements of arms | | |
| 1 – Flexed/Extended | Tense, straight legs; rigid and/or rapid extension/flexion | | |
| **Legs** | |  | |
| 0 – Relaxed/Restrained | No muscular rigidity, occasional random movements of legs | | |
| 1 – Flexed/Extended | Tense, straight legs; rigid and/or rapid extension/flexion | | |
| **State of Arousal** | |  | |
| 0 – Sleeping/Awake | Quiet, peaceful, sleeping, or alert random leg movement | | |
| 1 – Fussy | Alert, restless, thrashing | | |
| **TOTAL** | | |  |

Rater: ____________**DATA COLLECTION FORM (PHASE 2)**

**BASIC STUDY INFORMATION**

Patient ID: _____________

Date and time of study initiation: _____________ (YYYY-MM-DD) ____________ (HH:MM)

Date and time of study completion: _____________ (YYYY-MM-DD) ____________ (HH:MM)

**DEMOGRAPHICS**

| Year of birth: _______ | Sex: ☐ Male ☐ Female | Hours of life (at enrollment): ________ |
| --- | --- | --- |
| Gestational age (at birth): ______ weeks ______ days | | Birth weight: _________ grams |
| Diagnosis(es) at time of enrollment: | | |
| ☐ Perinatal asphyxia ☐ Anemia ☐ Apneas and bradycardias ☐ Bronchopulmonary dysplasia ☐ Hydrocephalus | | |
| ☐ Intraventricular hemorrhage ☐ Jaundice ☐ Necrotizing enterocolitis ☐ Other, specify: ___________________ | | |
| Treatments at enrollment: | | |
| ☐ Therapeutic hypothermia | | ☐ Continuous positive airway pressure (CPAP) |
| ☐ Conventional mechanical ventilation (CMV) | | ☐ High frequency ventilation (HFV) |
| ☐ Nasal intermittent positive pressure ventilation (NIPPV) | | ☐ Not applicable/healthy infants |
| Patient group: ☐ A ☐ B ☐ C ☐ D ☐ E ☐ F ☐ G ☐ H | | |

| A = healthy term infants in room air at enrollment  B = term infants with perinatal asphyxia undergoing therapeutic hypothermia at enrollment  C = healthy preterm infants in room air at enrollment  D = preterm infants on continuous positive airway pressure at enrollment  E = preterm infants on conventional mechanical ventilation at enrollment  F = preterm infants on high frequency ventilation at enrollment  G = preterm infants on nasal intermittent positive end expiratory pressure at enrollment  H = preterm infants on continuous positive airway pressure at enrollment |
| --- |

**HOURLY LOGS**

| Start Date: Start Time: End Date: End Time: | | | | | | | | | | | | | |
| --- | --- | --- | --- | --- | --- | --- | --- | --- | --- | --- | --- | --- | --- |
|  | baseline | 1h | 2h | 3h | 4h | 5h | 6h | 7h | 8h | 9h | 10h | 11h | 12h |
| Location of chest unit  (Center, R or L side) |  |  |  |  |  |  |  |  |  |  |  |  |  |
| Location of abdominal unit (Center, R or L side) |  |  |  |  |  |  |  |  |  |  |  |  |  |
| Location of limb unit  (R-right, L-left, H-hand/wrist, F-foot) |  |  |  |  |  |  |  |  |  |  |  |  |  |
| Number of conventional wires |  |  |  |  |  |  |  |  |  |  |  |  |  |
| Axillary temperature (°C) |  |  |  |  |  |  |  |  |  |  |  |  |  |
| Room temperature (°C) |  |  |  |  |  |  |  |  |  |  |  |  |  |
| Air temperature (°C) & humidity (%) - 15cm from neonate |  |  |  |  |  |  |  |  |  |  |  |  |  |
| Kangaroo care  (Y/N) |  |  |  |  |  |  |  |  |  |  |  |  |  |
| Nursing care  (Y/N) |  |  |  |  |  |  |  |  |  |  |  |  |  |
| Medications  (Write down any medication in use) |  |  |  |  |  |  |  |  |  |  |  |  |  |
| Phototherapy  (Y/N) |  |  |  |  |  |  |  |  |  |  |  |  |  |
| Type of ventilatory support (CPAP, CMV, HFV, NIPPV) |  |  |  |  |  |  |  |  |  |  |  |  |  |
| Neonate location  (incubator/crib) |  |  |  |  |  |  |  |  |  |  |  |  |  |
| Neonate position (s-supine, p-prone, side) |  |  |  |  |  |  |  |  |  |  |  |  |  |
| Sensor disconnections (N)/reasons* |  |  |  |  |  |  |  |  |  |  |  |  |  |
|  | 13h | 14h | 15h | 16h | 17h | 18h | 19h | 20h | 21h | 22h | 23h | 24h | 25h |
| Location of chest unit  (Center, R or L side) |  |  |  |  |  |  |  |  |  |  |  |  |  |
| Location of abdominal unit (Center, R or L side) |  |  |  |  |  |  |  |  |  |  |  |  |  |
| Location of limb unit  (R-right, L-left, H-hand/wrist, F-foot) |  |  |  |  |  |  |  |  |  |  |  |  |  |
| Number of conventional wires |  |  |  |  |  |  |  |  |  |  |  |  |  |
| Axillary temperature (°C) |  |  |  |  |  |  |  |  |  |  |  |  |  |
| Room temperature (°C) |  |  |  |  |  |  |  |  |  |  |  |  |  |
| Air temperature (°C) & humidity (%) - 15cm from neonate |  |  |  |  |  |  |  |  |  |  |  |  |  |
| Kangaroo care  (Y/N) |  |  |  |  |  |  |  |  |  |  |  |  |  |
| Nursing care  (Y/N) |  |  |  |  |  |  |  |  |  |  |  |  |  |
| Medications  (Write down any medication in use) |  |  |  |  |  |  |  |  |  |  |  |  |  |
| Phototherapy  (Y/N) |  |  |  |  |  |  |  |  |  |  |  |  |  |
| Type of ventilatory support (CPAP, CMV, HFV, NIPPV) |  |  |  |  |  |  |  |  |  |  |  |  |  |
| Neonate location  (incubator/crib) |  |  |  |  |  |  |  |  |  |  |  |  |  |
| Neonate position (s-supine, p-prone, side) |  |  |  |  |  |  |  |  |  |  |  |  |  |
| Sensor disconnections (N)/reasons* |  |  |  |  |  |  |  |  |  |  |  |  |  |
|  | 26h | 27h | 28h | 29h | 30h | 31h | 32h | 33h | 34h | 35h | 36h | 37h | 38h |
| Location of chest unit  (Center, R or L side) |  |  |  |  |  |  |  |  |  |  |  |  |  |
| Location of abdominal unit (Center, R or L side) |  |  |  |  |  |  |  |  |  |  |  |  |  |
| Location of limb unit  (R-right, L-left, H-hand/wrist, F-foot) |  |  |  |  |  |  |  |  |  |  |  |  |  |
| Number of conventional wires |  |  |  |  |  |  |  |  |  |  |  |  |  |
| Axillary temperature (°C) |  |  |  |  |  |  |  |  |  |  |  |  |  |
| Room temperature (°C) |  |  |  |  |  |  |  |  |  |  |  |  |  |
| Air temperature (°C) & humidity (%) - 15cm from neonate |  |  |  |  |  |  |  |  |  |  |  |  |  |
| Kangaroo care  (Y/N) |  |  |  |  |  |  |  |  |  |  |  |  |  |
| Nursing care  (Y/N) |  |  |  |  |  |  |  |  |  |  |  |  |  |
| Medications  (Write down any medication in use) |  |  |  |  |  |  |  |  |  |  |  |  |  |
| Phototherapy  (Y/N) |  |  |  |  |  |  |  |  |  |  |  |  |  |
| Type of ventilatory support (CPAP, CMV, HFV, NIPPV) |  |  |  |  |  |  |  |  |  |  |  |  |  |
| Neonate location  (incubator/crib) |  |  |  |  |  |  |  |  |  |  |  |  |  |
| Neonate position (s-supine, p-prone, side) |  |  |  |  |  |  |  |  |  |  |  |  |  |
| Sensor disconnections (N)/reasons* |  |  |  |  |  |  |  |  |  |  |  |  |  |
|  | 39h | 40h | 41h | 42h | 43h | 44h | 45h | 46h | 47h | 48h | 49h | 50h | 51h |
| Location of chest unit  (Center, R or L side) |  |  |  |  |  |  |  |  |  |  |  |  |  |
| Location of abdominal unit (Center, R or L side) |  |  |  |  |  |  |  |  |  |  |  |  |  |
| Location of limb unit  (R-right, L-left, H-hand/wrist, F-foot) |  |  |  |  |  |  |  |  |  |  |  |  |  |
| Number of conventional wires |  |  |  |  |  |  |  |  |  |  |  |  |  |
| Axillary temperature (°C) |  |  |  |  |  |  |  |  |  |  |  |  |  |
| Room temperature (°C) |  |  |  |  |  |  |  |  |  |  |  |  |  |
| Air temperature (°C) & humidity (%) - 15cm from neonate |  |  |  |  |  |  |  |  |  |  |  |  |  |
| Kangaroo care  (Y/N) |  |  |  |  |  |  |  |  |  |  |  |  |  |
| Nursing care  (Y/N) |  |  |  |  |  |  |  |  |  |  |  |  |  |
| Medications  (Write down any medication in use) |  |  |  |  |  |  |  |  |  |  |  |  |  |
| Phototherapy  (Y/N) |  |  |  |  |  |  |  |  |  |  |  |  |  |
| Type of ventilatory support (CPAP, CMV, HFV, NIPPV) |  |  |  |  |  |  |  |  |  |  |  |  |  |
| Neonate location  (incubator/crib) |  |  |  |  |  |  |  |  |  |  |  |  |  |
| Neonate position (s-supine, p-prone, side) |  |  |  |  |  |  |  |  |  |  |  |  |  |
| Sensor disconnections (N)/reasons* |  |  |  |  |  |  |  |  |  |  |  |  |  |
|  | 52h | 53h | 54h | 55h | 56h | 57h | 58h | 59h | 60h | 61h | 62h | 63h | 64h |
| Location of chest unit  (Center, R or L side) |  |  |  |  |  |  |  |  |  |  |  |  |  |
| Location of abdominal unit (Center, R or L side) |  |  |  |  |  |  |  |  |  |  |  |  |  |
| Location of limb unit  (R-right, L-left, H-hand/wrist, F-foot) |  |  |  |  |  |  |  |  |  |  |  |  |  |
| Number of conventional wires |  |  |  |  |  |  |  |  |  |  |  |  |  |
| Axillary temperature (°C) |  |  |  |  |  |  |  |  |  |  |  |  |  |
| Room temperature (°C) |  |  |  |  |  |  |  |  |  |  |  |  |  |
| Air temperature (°C) & humidity (%) - 15cm from neonate |  |  |  |  |  |  |  |  |  |  |  |  |  |
| Kangaroo care  (Y/N) |  |  |  |  |  |  |  |  |  |  |  |  |  |
| Nursing care  (Y/N) |  |  |  |  |  |  |  |  |  |  |  |  |  |
| Medications  (Write down any medication in use) |  |  |  |  |  |  |  |  |  |  |  |  |  |
| Phototherapy  (Y/N) |  |  |  |  |  |  |  |  |  |  |  |  |  |
| Type of ventilatory support (CPAP, CMV, HFV, NIPPV) |  |  |  |  |  |  |  |  |  |  |  |  |  |
| Neonate location  (incubator/crib) |  |  |  |  |  |  |  |  |  |  |  |  |  |
| Neonate position (s-supine, p-prone, side) |  |  |  |  |  |  |  |  |  |  |  |  |  |
| Sensor disconnections (N)/reasons* |  |  |  |  |  |  |  |  |  |  |  |  |  |
|  | 65h | 66h | 67h | 68h | 69h | 70h | 71h | 72h | 73h | 74h | 75h | 76h | 77h |
| Location of chest unit  (Center, R or L side) |  |  |  |  |  |  |  |  |  |  |  |  |  |
| Location of abdominal unit (Center, R or L side) |  |  |  |  |  |  |  |  |  |  |  |  |  |
| Location of limb unit  (R-right, L-left, H-hand/wrist, F-foot) |  |  |  |  |  |  |  |  |  |  |  |  |  |
| Number of conventional wires |  |  |  |  |  |  |  |  |  |  |  |  |  |
| Axillary temperature (°C) |  |  |  |  |  |  |  |  |  |  |  |  |  |
| Room temperature (°C) |  |  |  |  |  |  |  |  |  |  |  |  |  |
| Air temperature (°C) & humidity (%) - 15cm from neonate |  |  |  |  |  |  |  |  |  |  |  |  |  |
| Kangaroo care  (Y/N) |  |  |  |  |  |  |  |  |  |  |  |  |  |
| Nursing care  (Y/N) |  |  |  |  |  |  |  |  |  |  |  |  |  |
| Medications  (Write down any medication in use) |  |  |  |  |  |  |  |  |  |  |  |  |  |
| Phototherapy  (Y/N) |  |  |  |  |  |  |  |  |  |  |  |  |  |
| Type of ventilatory support (CPAP, CMV, HFV, NIPPV) |  |  |  |  |  |  |  |  |  |  |  |  |  |
| Neonate location  (incubator/crib) |  |  |  |  |  |  |  |  |  |  |  |  |  |
| Neonate position (s-supine, p-prone, side) |  |  |  |  |  |  |  |  |  |  |  |  |  |
| Sensor disconnections (N)/reasons* |  |  |  |  |  |  |  |  |  |  |  |  |  |
|  | 78h | 79h | 80h | 81h | 82h | 83h | 84h | 85h | 86h | 87h | 88h | 89h | 90h |
| Location of chest unit  (Center, R or L side) |  |  |  |  |  |  |  |  |  |  |  |  |  |
| Location of abdominal unit (Center, R or L side) |  |  |  |  |  |  |  |  |  |  |  |  |  |
| Location of limb unit  (R-right, L-left, H-hand/wrist, F-foot) |  |  |  |  |  |  |  |  |  |  |  |  |  |
| Number of conventional wires |  |  |  |  |  |  |  |  |  |  |  |  |  |
| Axillary temperature (°C) |  |  |  |  |  |  |  |  |  |  |  |  |  |
| Room temperature (°C) |  |  |  |  |  |  |  |  |  |  |  |  |  |
| Air temperature (°C) & humidity (%) - 15cm from neonate |  |  |  |  |  |  |  |  |  |  |  |  |  |
| Kangaroo care  (Y/N) |  |  |  |  |  |  |  |  |  |  |  |  |  |
| Nursing care  (Y/N) |  |  |  |  |  |  |  |  |  |  |  |  |  |
| Medications  (Write down any medication in use) |  |  |  |  |  |  |  |  |  |  |  |  |  |
| Phototherapy  (Y/N) |  |  |  |  |  |  |  |  |  |  |  |  |  |
| Type of ventilatory support (CPAP, CMV, HFV, NIPPV) |  |  |  |  |  |  |  |  |  |  |  |  |  |
| Neonate location  (incubator/crib) |  |  |  |  |  |  |  |  |  |  |  |  |  |
| Neonate position (s-supine, p-prone, side) |  |  |  |  |  |  |  |  |  |  |  |  |  |
| Sensor disconnections (N)/reasons* |  |  |  |  |  |  |  |  |  |  |  |  |  |
|  | 91h | 92h | 93h | 94h | 95h | 96h |  |  |  |  |  |  |  |
| Location of chest unit  (Center, R or L side) |  |  |  |  |  |  |  |  |  |  |  |  |  |
| Location of abdominal unit (Center, R or L side) |  |  |  |  |  |  |  |  |  |  |  |  |  |
| Location of limb unit  (R-right, L-left, H-hand/wrist, F-foot) |  |  |  |  |  |  |  |  |  |  |  |  |  |
| Number of conventional wires |  |  |  |  |  |  |  |  |  |  |  |  |  |
| Axillary temperature (°C) |  |  |  |  |  |  |  |  |  |  |  |  |  |
| Room temperature (°C) |  |  |  |  |  |  |  |  |  |  |  |  |  |
| Air temperature (°C) & humidity (%) - 15cm from neonate |  |  |  |  |  |  |  |  |  |  |  |  |  |
| Kangaroo care  (Y/N) |  |  |  |  |  |  |  |  |  |  |  |  |  |
| Nursing care  (Y/N) |  |  |  |  |  |  |  |  |  |  |  |  |  |
| Medications  (Write down any medication in use) |  |  |  |  |  |  |  |  |  |  |  |  |  |
| Phototherapy  (Y/N) |  |  |  |  |  |  |  |  |  |  |  |  |  |
| Type of ventilatory support (CPAP, CMV, HFV, NIPPV) |  |  |  |  |  |  |  |  |  |  |  |  |  |
| Neonate location  (incubator/crib) |  |  |  |  |  |  |  |  |  |  |  |  |  |
| Neonate position (s-supine, p-prone, side) |  |  |  |  |  |  |  |  |  |  |  |  |  |
| Sensor disconnections (N)/reasons* |  |  |  |  |  |  |  |  |  |  |  |  |  |

*A = x-ray, B = bath, C = cleaning, D = tests, E = transport, F = other

**PHOTOS OF THE SKIN AT SENSOR PLACEMENT SITES**

(place “✓” if done)

|  | **Chest:** | | | |
| --- | --- | --- | --- | --- |
| ☐ Baseline (0h) | ☐ Sensor replacement #1  Date:  Time: | ☐ Sensor replacement #2  Date:  Time: | ☐ Sensor replacement #3  Date:  Time: | ☐ 96h |
|  | **Abdomen:** | | | |
| ☐ Baseline (0h) | ☐ Sensor replacement #1  Date:  Time: | ☐ Sensor replacement #2  Date:  Time: | ☐ Sensor replacement #3  Date:  Time: | ☐ 96h |
|  | **Limb:** | | | |
| ☐ Baseline (0h) | ☐ Sensor replacement #1  Date:  Time: | ☐ Sensor replacement #2  Date:  Time: | ☐ Sensor replacement #3  Date:  Time: | ☐ 96h |

**NEONATAL INFANT PAIN SCALE**

Sensor replacement #1 – Time: ____________ Rater: ____________

| **Pain Assessment** | | Score |
| --- | --- | --- |
| **Facial Expression** | |  |
| 0 – Relaxed muscles | Restful face, neutral expression | |
| 1 – Grimace | Tight facial muscles, furrowed brow, chin, jaw, (negative facial expression – nose, mouth, brow) | |
| **Cry** | |  |
| 0 – No cry | Quiet, not crying | |
| 1 - Whimper | Mild moaning, intermittent | |
| 2 – Vigorous Cry | Loud scream; rising, shrill, continuous (Note: silent cry may be scored if baby is intubated as evidenced by obvious mouth and facial movement.) | |
| **Breathing Patterns** | |  |
| 0 – Relaxed | Usual pattern for this infant | |
| 1 – Change in Breathing | Indrawing, irregular, faster than usual, gagging, breath holding | |
| **Arms** | |  |
| 0 – Relaxed/Restrained | No muscular rigidity, occasional random movements of arms | |
| 1 – Flexed/Extended | Tense, straight legs; rigid and/or rapid extension/flexion | |
| **Legs** | |  |
| 0 – Relaxed/Restrained | No muscular rigidity, occasional random movements of legs | |
| 1 – Flexed/Extended | Tense, straight legs; rigid and/or rapid extension/flexion | |
| **State of Arousal** | |  |
| 0 – Sleeping/Awake | Quiet, peaceful, sleeping, or alert random leg movement | |
| 1 – Fussy | Alert, restless, thrashing | |
| **TOTAL** | |  |

Sensor replacement #2 – Time: ____________Rater: ____________

| **Pain Assessment** | | Score |
| --- | --- | --- |
| **Facial Expression** | |  |
| 0 – Relaxed muscles | Restful face, neutral expression | |
| 1 – Grimace | Tight facial muscles, furrowed brow, chin, jaw, (negative facial expression – nose, mouth, brow) | |
| **Cry** | |  |
| 0 – No cry | Quiet, not crying | |
| 1 - Whimper | Mild moaning, intermittent | |
| 2 – Vigorous Cry | Loud scream; rising, shrill, continuous (Note: silent cry may be scored if baby is intubated as evidenced by obvious mouth and facial movement.) | |
| **Breathing Patterns** | |  |
| 0 – Relaxed | Usual pattern for this infant | |
| 1 – Change in Breathing | Indrawing, irregular, faster than usual, gagging, breath holding | |
| **Arms** | |  |
| 0 – Relaxed/Restrained | No muscular rigidity, occasional random movements of arms | |
| 1 – Flexed/Extended | Tense, straight legs; rigid and/or rapid extension/flexion | |
| **Legs** | |  |
| 0 – Relaxed/Restrained | No muscular rigidity, occasional random movements of legs | |
| 1 – Flexed/Extended | Tense, straight legs; rigid and/or rapid extension/flexion | |
| **State of Arousal** | |  |
| 0 – Sleeping/Awake | Quiet, peaceful, sleeping, or alert random leg movement | |
| 1 – Fussy | Alert, restless, thrashing | |
| **TOTAL** | |  |

Sensor replacement #3 – Time: ____________ Rater: ____________

| **Pain Assessment** | | Score |
| --- | --- | --- |
| **Facial Expression** | |  |
| 0 – Relaxed muscles | Restful face, neutral expression | |
| 1 – Grimace | Tight facial muscles, furrowed brow, chin, jaw, (negative facial expression – nose, mouth, brow) | |
| **Cry** | |  |
| 0 – No cry | Quiet, not crying | |
| 1 - Whimper | Mild moaning, intermittent | |
| 2 – Vigorous Cry | Loud scream; rising, shrill, continuous (Note: silent cry may be scored if baby is intubated as evidenced by obvious mouth and facial movement.) | |
| **Breathing Patterns** | |  |
| 0 – Relaxed | Usual pattern for this infant | |
| 1 – Change in Breathing | Indrawing, irregular, faster than usual, gagging, breath holding | |
| **Arms** | |  |
| 0 – Relaxed/Restrained | No muscular rigidity, occasional random movements of arms | |
| 1 – Flexed/Extended | Tense, straight legs; rigid and/or rapid extension/flexion | |
| **Legs** | |  |
| 0 – Relaxed/Restrained | No muscular rigidity, occasional random movements of legs | |
| 1 – Flexed/Extended | Tense, straight legs; rigid and/or rapid extension/flexion | |
| **State of Arousal** | |  |
| 0 – Sleeping/Awake | Quiet, peaceful, sleeping, or alert random leg movement | |
| 1 – Fussy | Alert, restless, thrashing | |
| **TOTAL** | |  |

Final sensor removal (96h) – Time: ____________ Rater: ____________

| **Pain Assessment** | | Score |
| --- | --- | --- |
| **Facial Expression** | |  |
| 0 – Relaxed muscles | Restful face, neutral expression | |
| 1 – Grimace | Tight facial muscles, furrowed brow, chin, jaw, (negative facial expression – nose, mouth, brow) | |
| **Cry** | |  |
| 0 – No cry | Quiet, not crying | |
| 1 - Whimper | Mild moaning, intermittent | |
| 2 – Vigorous Cry | Loud scream; rising, shrill, continuous (Note: silent cry may be scored if baby is intubated as evidenced by obvious mouth and facial movement.) | |
| **Breathing Patterns** | |  |
| 0 – Relaxed | Usual pattern for this infant | |
| 1 – Change in Breathing | Indrawing, irregular, faster than usual, gagging, breath holding | |
| **Arms** | |  |
| 0 – Relaxed/Restrained | No muscular rigidity, occasional random movements of arms | |
| 1 – Flexed/Extended | Tense, straight legs; rigid and/or rapid extension/flexion | |
| **Legs** | |  |
| 0 – Relaxed/Restrained | No muscular rigidity, occasional random movements of legs | |
| 1 – Flexed/Extended | Tense, straight legs; rigid and/or rapid extension/flexion | |
| **State of Arousal** | |  |
| 0 – Sleeping/Awake | Quiet, peaceful, sleeping, or alert random leg movement | |
| 1 – Fussy | Alert, restless, thrashing | |
| **TOTAL** | |  |

**RESPIRATION MEASUREMENTS USING UNCALIBRATED RIP BELTS**

**Day 1**: From ____________ (HH:MM) to ____________ (HH:MM)

**Day 2**: From ____________ (HH:MM) to ____________ (HH:MM)

**Day 3**: From ____________ (HH:MM) to ____________ (HH:MM)

**Day 4**: From ____________ (HH:MM) to ____________ (HH:MM)

**ADVERSE EVENTS**

☐ Skin irritation/redness

If observed, complete section below:

| Onset date: ________________ (YYYY-MM-DD) | Location: ________________ |
| --- | --- |
| Severity: ☐ Mild ☐ Moderate ☐ Severe | Treatment required? ☐ Yes ☐ No |
| Relationship to study sensors: ☐ Related ☐ Suspected ☐ Not suspected | |
| Outcome: ☐ Recovered ☐ Improving ☐ Unchanged ☐ End of study participation | |

☐ Skin erosions

If observed, complete section below:

| Onset date: ________________ (YYYY-MM-DD) | Location: ________________ |
| --- | --- |
| Severity: ☐ Mild ☐ Moderate ☐ Severe | Treatment required? ☐ Yes ☐ No |
| Relationship to study sensors: ☐ Related ☐ Suspected ☐ Not suspected | |
| Outcome: ☐ Recovered ☐ Improving ☐ Unchanged ☐ End of study participation | |

☐ Skin bleeding

If observed, complete section below:

| Onset date: ________________ (YYYY-MM-DD) | Location: ________________ |
| --- | --- |
| Severity: ☐ Mild ☐ Moderate ☐ Severe | Treatment required? ☐ Yes ☐ No |
| Relationship to study sensors: ☐ Related ☐ Suspected ☐ Not suspected | |
| Outcome: ☐ Recovered ☐ Improving ☐ Unchanged ☐ End of study participation | |

☐ Other, specify: ___________________

**ADVERSE EVENTS**

☐ Skin irritation/redness

If observed, complete section below:

| Onset date: ________________ (YYYY-MM-DD) | Location: ________________ |
| --- | --- |
| Severity: ☐ Mild ☐ Moderate ☐ Severe | Treatment required? ☐ Yes ☐ No |
| Relationship to study sensors: ☐ Related ☐ Suspected ☐ Not suspected | |
| Outcome: ☐ Recovered ☐ Improving ☐ Unchanged ☐ End of study participation | |

☐ Skin erosions

If observed, complete section below:

| Onset date: ________________ (YYYY-MM-DD) | Location: ________________ |
| --- | --- |
| Severity: ☐ Mild ☐ Moderate ☐ Severe | Treatment required? ☐ Yes ☐ No |
| Relationship to study sensors: ☐ Related ☐ Suspected ☐ Not suspected | |
| Outcome: ☐ Recovered ☐ Improving ☐ Unchanged ☐ End of study participation | |

☐ Skin bleeding

If observed, complete section below:

| Onset date: ________________ (YYYY-MM-DD) | Location: ________________ |
| --- | --- |
| Severity: ☐ Mild ☐ Moderate ☐ Severe | Treatment required? ☐ Yes ☐ No |
| Relationship to study sensors: ☐ Related ☐ Suspected ☐ Not suspected | |
| Outcome: ☐ Recovered ☐ Improving ☐ Unchanged ☐ End of study participation | |

☐ Other, specify: ___________________
