# Supplementary material for: The use of wireless sensors in the neonatal intensive care unit: a study protocol
Source: PeerJ. 2023 Jun 27;11:e15578. doi: 10.7717/peerj.15578 (PMC10312156; doi:10.7717/peerj.15578)
Supplement: Supplemental Information 4 — List of abbreviations used to annotate different types of clinical events and care which may occur during study monitoring periods and impact data quality and values. [file peerj-11-15578-s004.docx]

|  | **CODE** | **Reason** |
| --- | --- | --- |
| **Sensors /BioDash Related** | AF | Biodash Application Failure/Malfunction |
|  | CP-ECG | Change of ECG probes |
|  | CP-RR | Change respiratory probe |
|  | CP-SpO2 | Change of SpO2 probe |
|  | CP-T | Change of temperature probe |
|  | PS | Poor Sensor Signal |
|  | SR-adj | Sensor readjustment |
|  | SR-rep | Sensor replacement |
|  | -SX | Sensor Removal |
| **Routine Care + Behaviors** | B | Bathing |
|  | BT | Blood Test (via heel stick or other local acute extraction method) |
|  | C | Crying |
|  | DC | Diaper change |
|  | KC | Kangaroo Care |
|  | PP | Prone positioning |
|  | RC | Routine care (other) **specify |
|  | SL | Sleeping |
|  | SP | Supine position |
| **Feeding** | BF | Breastfeeding |
|  | FB | Feeding by bottle |
|  | G | Gavage |
| **Respiratory & Cardiac** | AC | Airway Care |
|  | CPAP | Continuous positive air pressure (specify type) |
|  | CRE | Cardiorespiratory Event (requiring nurse intervention) |
|  | EX | Extubation |
|  | I | Intubation |
|  | MV | Mechanical Ventilation |
|  | NC | Nasal canula |
|  | NIPPV | Nasal Intermittent Positive Pressure Ventilation |
| **Clinical Events** | CS | Clinical Seizure |
|  | F | Fever (temperature above 37.8 C) |
|  | H | Hypothermia (temperature below 36 C) |
|  | SaEEG | Seizure - aEEG |
|  | SEEG | Seizure - EEG |
| **Treatments & Procedures** | AL | Arterial Line |
|  | BTR | Blood Transfusion |
|  | IV | Intravenous line |
|  | MA | Medication Administration (specify medication) |
|  | NT | Nasogastric tube |
|  | PS | Painful stimulus (i.e. painful procedure) |
|  | PT | Phototherapy |
|  | T | Transport/transfer |
|  | TH | Therapeutic Hypothermia |
|  | U | Ultrasound |
|  | XR | X-Ray |
| **Other** | O | Other event, specify |
