# Supplementary material for: The use of wireless sensors in the neonatal intensive care unit: a study protocol
Source: PeerJ. 2023 Jun 27;11:e15578. doi: 10.7717/peerj.15578 (PMC10312156; doi:10.7717/peerj.15578)
Supplement: Supplemental Information 5 — Number of data points obtained per participant for each signal monitored based on duration of monitoring and sampling rate of monitoring technology. [file peerj-11-15578-s005.docx]

| **Device** | **Philips Intellivue MX450** |  | **Wireless ANNE™ Monitoring System** |  |
| --- | --- | --- | --- | --- |
| **STUDY Phase** | **Phase 1** | **Phase 2** | **Phase 1** | **Phase 2** |
| **ECG** | 1.44 × 10^7 | 1.728 × 10^8 | 7.3657289 × 10^6 | 8.83887468 × 10^7 |
| **PPG** | 4.32 × 10^7 | 4.32 × 10^7 | 1.842610365 × 10^6 | 2.211132438 × 10^7 |
| **Resp signal** | 1.8 × 10^6 | 2.16 × 10^7 | 9.216 × 10^5 | 1.10592 × 10^7 |
| **HR** | 2.8125 × 10^4 | 3.375 × 10^5 | 2.88 × 10^4 | 3.456 × 10^5 |
| **SpO2** | 2.8125 × 10^5 | 3.375 ×10^6 | 2.88 × 10^4 | 3.456 × 10^5 |
| **RR** | 2.8125 × 10^6 | 3.375 × 10^7 | 2.88 x10^4 | 3.456 × 10^5 |
| **Temperature (axial & chest respectively)** | 8 | 9.6 x 10^1 | 7.2 × 10^3 | 8.64 × 10^4 |
| **Skin temp. limb** | N/A | N/A | 7.2 × 10^3 | 8.64 × 10^4 |
| **3-axis Accel.** | N/A | N/A | 1.2 × 10^7 | 1.44 × 10^8 |
| **Total** | 1.9884383 × 10^7 | 2.38612596 × 10^8 | 2.223073926 ×10^7 | 2.667688712 × 10^8 |
