# Supplementary material for: The use of wireless sensors in the neonatal intensive care unit: a study protocol
Source: PeerJ. 2023 Jun 27;11:e15578. doi: 10.7717/peerj.15578 (PMC10312156; doi:10.7717/peerj.15578)
Supplement: Supplemental Information 6 — Total number of data points obtained across all participants participant for each signal monitored based on duration of monitoring and sampling rate of monitoring technology. [file peerj-11-15578-s006.docx]

| **Device** | **Philips IntelliVue MX450** |  | **Wireless ANNE™ Monitoring System** |  |
| --- | --- | --- | --- | --- |
| **STUDY Phase** | **Phase 1** | **Phase 2** | **Phase 1** | **Phase 2** |
| **ECG** | 3.456 × 10^8 | 4.1472 × 10^9 | 1.767774936 × 10^8 | 2.121329923 × 10^9 |
| **PPG** | 8.64 × 10^7 | 1.0368 × 10^9 | 4.422264875 × 10^7 | 5.30671785 × 10^8 |
| **Resp signal** | 4.32 × 10^7 | 5.184 × 10^8 | 2.21184 × 10^7 | 2.654208 × 10^8 |
| **HR** | 6.75 × 10^5 | 8.1 × 10^6 | 6.912 × 10^5 | 8.2944 × 10^6 |
| **SpO2** | 6.75 × 10^5 | 8.1 × 10^6 | 6.912 × 10^5 | 8.2944 × 10^6 |
| **RR** | 6.75 × 10^5 | 8.1 × 10^6 | 6.912 × 10^5 | 8.2944 × 10^6 |
| **Temperature (axial & chest respectively)** | 1.921.92 × 10^2 | 2.304 × 10^3 | 1.728 × 10^5 | 2.0736 × 10^6 |
| **Skin temp. limb** | N/A | N/A | 1.728 × 10^5 | 2.0736 × 10^6 |
| **3-axis Accel.** | N/A | N/A | 2.88 × 10^8 | 3.456 × 10^9 |
| **Total** | 4.77225192 × 10^8 | 5.726702304 × 10^9 | 5.335377424 × 10^8 | 6.402452908 × 10^9 |
